# Supplementary material for: Pulmonary mucoid Pseudomonas aeruginosa infection and association with higher species richness and stronger inflammatory immune response
Source: Microbiol Spectr. 2026 Mar 23;14(5):e02295-25. doi: 10.1128/spectrum.02295-25 (PMC13141930; doi:10.1128/spectrum.02295-25)
Supplement: Supplemental material — Table S1; Fig. S1 to S3. [file spectrum.02295-25-s0001.pdf]

Supplement Materials

Content

1. Table S1 The demographic and clinical characteristics of the patients
2. Figure S1 16S rDNA sequence length distribution
3. Figure S2 Taxonomic annotation of species
4. Figure S3 β diversity based on NMDS

Table S1 The demographic and clinical characteristics of the patients

Characteristics	MPA（n=20）	NMPA（n=40）	Statistic Value	*P*
Age	65.55±14.17	66.78±14.81	t=0.306	0.76
Sex（Male/Female）	6/14	19/21	X^2=6.56	0.63
Smoking	8（40%）	15（37.5%）	χ^2=0.18	0.65
Drinking	5（25%）	17（42.5%）	χ^2=0.38	0.53
Hypertension	10（50%）	21（52.5%）	X^2=0.33	0.85
Diabetes	7（35%）	7（17.5%）	X^2=2.28	0.13
Cerebrovascular diseases	5（25%）	24（60%）	X^2=6.54	0.42
Heart disease	4（20%）	9（22.5%）	X^2=0.05	0.34
White blood cell count	8.21(6.30,9.61)	7.92(6.85, 10.84)	Z= - 0.45	0.66
Neutrophil count	4.57(3.22,7.26)	5.54(3.81,7.64)	Z= -1.25	0.21
Percentage of neutrophils	64(54.34,74.05)	67.2(61.45,77.65)	Z=-1.08	0.28
Lymphocyte count	1.38(0.95,2.25)	1.61(1.02,2.30)	Z=-0.03	0.98
Percentage of lymphocytes	23.95(13.7,33.65)	20.6(11.55,24.75)	Z=-1.23	0.22
Hypersensitive C-reactive protein	15.99(6.99,60.47)	15.61(8.58,34.14)	Z=-0.20	0.84

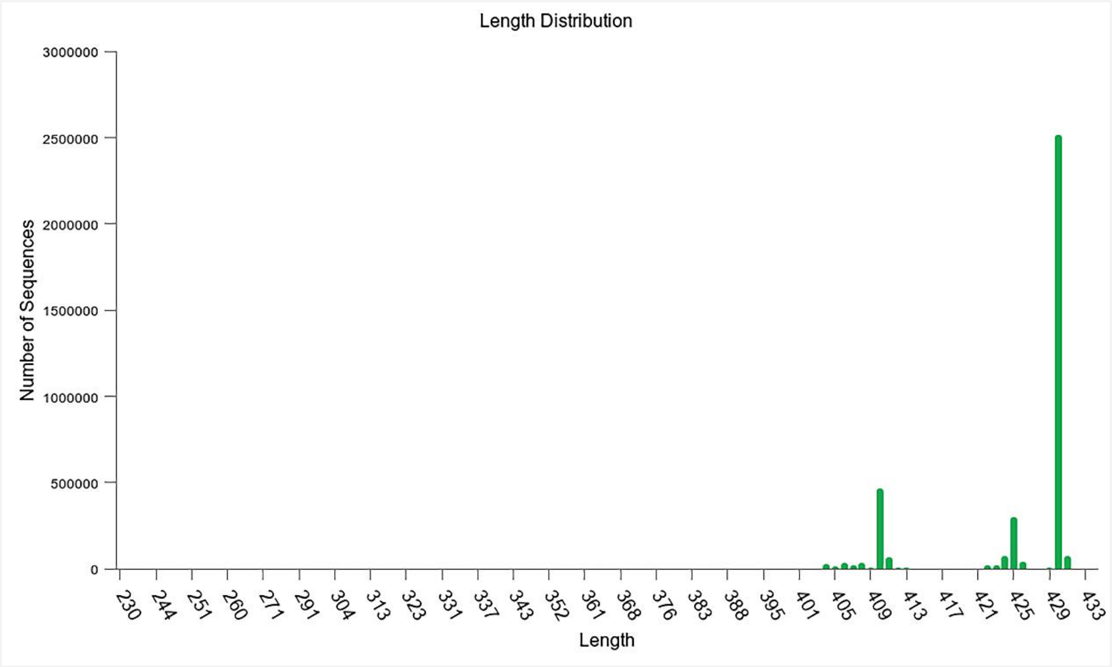

Figure S1 16S rDNA sequence length distribution

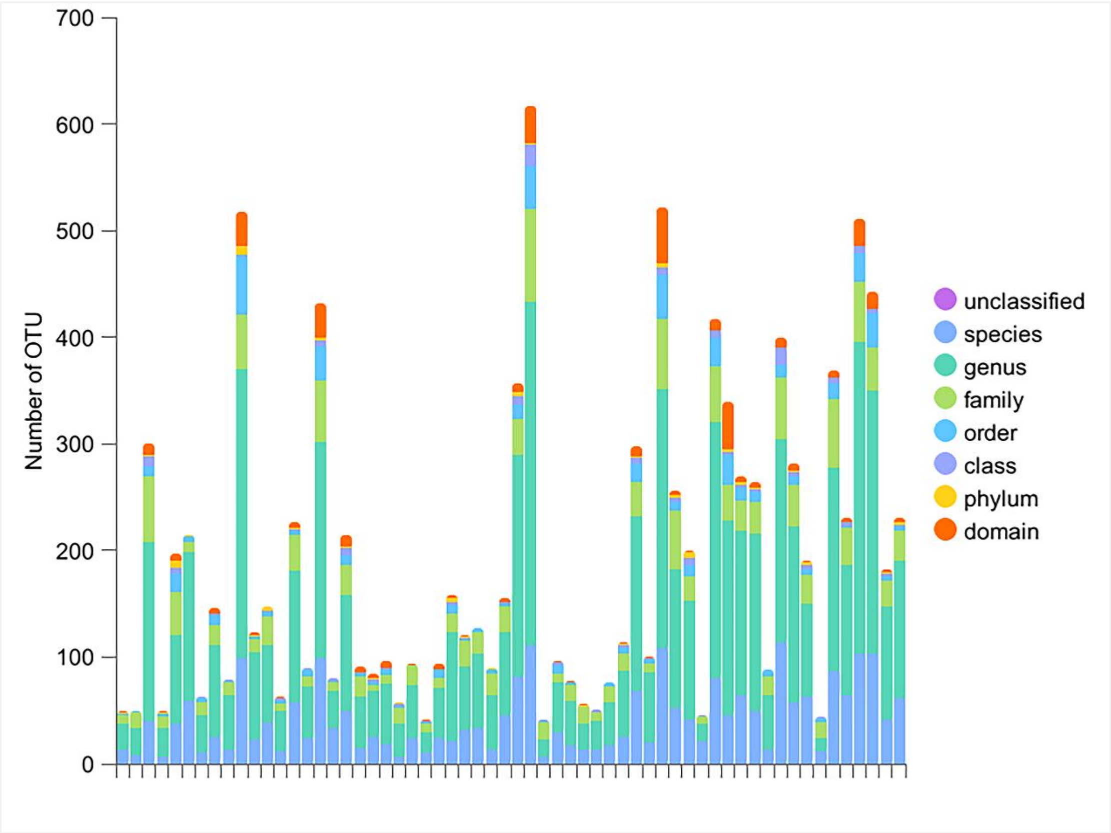

Figure S2 Taxonomic annotation of species

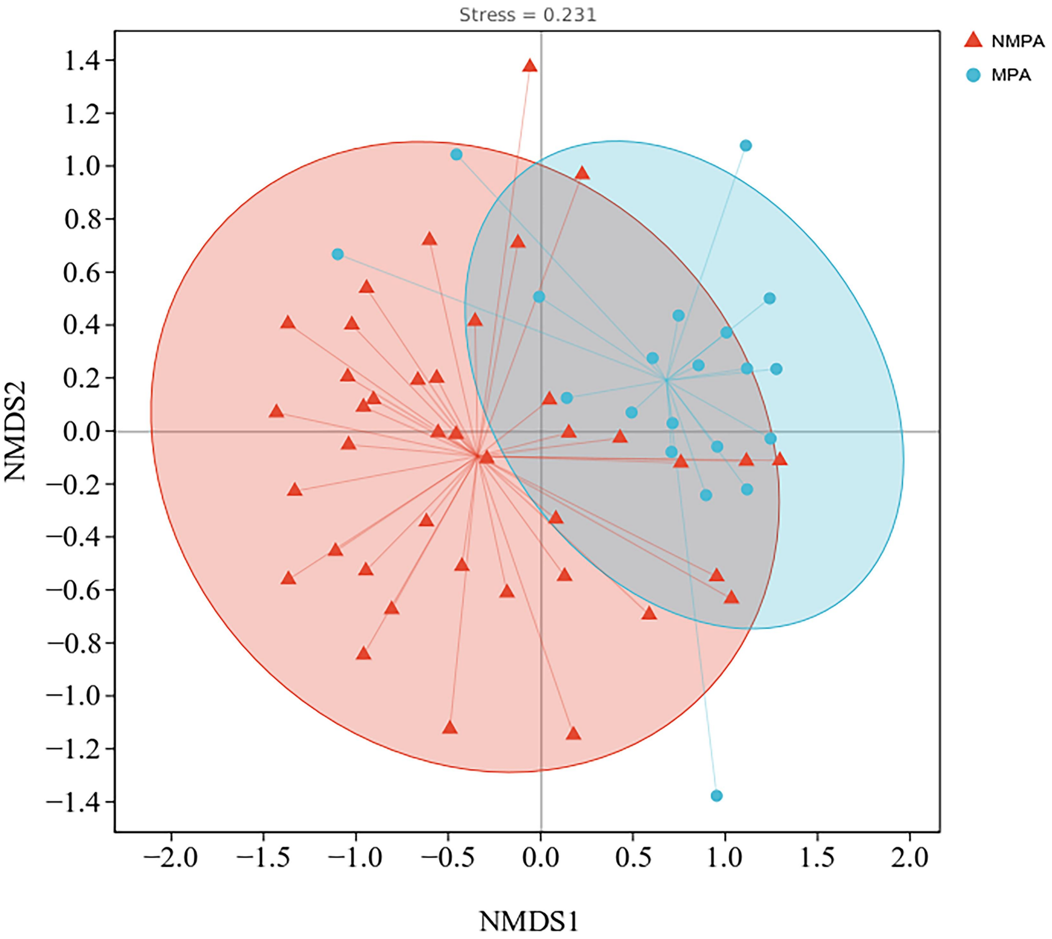

Figure S3 β diversity based on NMDS
